# Prevalence of Health Misinformation on Social Media—Challenges and Mitigation Before, During, and Beyond the COVID-19 Pandemic: Scoping Literature Review

**DOI:** 10.2196/38786

**Published:** 2024-08-19

**Authors:** Dhouha Kbaier, Annemarie Kane, Mark McJury, Ian Kenny

**Affiliations:** 1 School of Computing and Communications The Open University Milton Keynes United Kingdom; 2 Faculty of Arts and Social Sciences The Open University Milton Keynes United Kingdom; 3 School of Physical Sciences The Open University Milton Keynes United Kingdom

**Keywords:** health misinformation, online health communities, vaccine hesitancy, social media, health professionals, public health, COVID-19, intervention, antivaxxers

## Abstract

**Background:**

This scoping review accompanies our research study “The Experience of Health Professionals With Misinformation and Its Impact on Their Job Practice: Qualitative Interview Study.” It surveys online health misinformation and is intended to provide an understanding of the communication context in which health professionals must operate.

**Objective:**

Our objective was to illustrate the impact of social media in introducing additional sources of misinformation that impact health practitioners’ ability to communicate effectively with their patients. In addition, we considered how the level of knowledge of practitioners mitigated the effect of misinformation and additional stress factors associated with dealing with outbreaks, such as the COVID-19 pandemic, that affect communication with patients.

**Methods:**

This study used a 5-step scoping review methodology following Arksey and O’Malley’s methodology to map relevant literature published in English between January 2012 and March 2024, focusing on health misinformation on social media platforms. We defined health misinformation as a false or misleading health-related claim that is not based on valid evidence or scientific knowledge. Electronic searches were performed on PubMed, Scopus, Web of Science, and Google Scholar. We included studies on the extent and impact of health misinformation in social media, mitigation strategies, and health practitioners’ experiences of confronting health misinformation. Our independent reviewers identified relevant articles for data extraction.

**Results:**

Our review synthesized findings from 70 sources on online health misinformation. It revealed a consensus regarding the significant problem of health misinformation disseminated on social network platforms. While users seek trustworthy sources of health information, they often lack adequate health and digital literacies, which is exacerbated by social and economic inequalities. Cultural contexts influence the reception of such misinformation, and health practitioners may be vulnerable, too. The effectiveness of online mitigation strategies like user correction and automatic detection are complicated by malicious actors and politicization. The role of health practitioners in this context is a challenging one. Although they are still best placed to combat health misinformation, this review identified stressors that create barriers to their abilities to do this well. Investment in health information management at local and global levels could enhance their capacity for effective communication with patients.

**Conclusions:**

This scoping review underscores the significance of addressing online health misinformation, particularly in the postpandemic era. It highlights the necessity for a collaborative global interdisciplinary effort to ensure equitable access to accurate health information, thereby empowering health practitioners to effectively combat the impact of online health misinformation. Academic research will need to be disseminated into the public domain in a way that is accessible to the public. Without equipping populations with health and digital literacies, the prevalence of online health misinformation will continue to pose a threat to global public health efforts.

## Introduction

The global adoption of the internet has made health information more accessible, and the development of digital technology has enabled its rapid dissemination. However, the internet has also made possible the dissemination of false and misleading health misinformation and disinformation, with negative consequences, including the potential to exacerbate health inequalities. Health practitioners have found themselves at the forefront of communicating with patients who have taken on board health misinformation in the context of its proliferation on the web. This paper (associated with the study by Ismail et al [1]) surveyed the current literature concerning online health misinformation to establish the extent and scope of the problem, with special reference to the needs of health practitioners in their efforts to mitigate its impact. Several studies have established useful definitions of the terms *misinformation* and *disinformation* and distinctions between them. Misinformation has been defined as information that is not supported by evidence and contradicts the best-supported evidence available [2,3]. Wang et al [4] made a further distinction between online misinformation and disinformation, in particular on social media platforms. For Wang et al [4], misinformation is information that is not known to be false and is shared without malice. By contrast, disinformation involves the knowing and sharing of false information with the purpose of causing harm. This paper follows the distinctions of Wang et al [4] to use the terms *misinformation* and *disinformation* as appropriate.

It is important to acknowledge at the outset that digital technology in health and social contexts presents both risks and opportunities for equity among different information audiences [5]. However, there has recently been a change in the reception and assessment of the role of the internet, social media in particular, among researchers, even predating the COVID-19 pandemic. In the early days of social media, researchers largely identified prosocial and altruistic uses of social media platforms such as Facebook and Twitter by the public. However, considerable disquiet about the impact of social media and its potential for the spread of “fake news” and the amplification of conspiracy theories has displaced the more positive evaluation that was apparent when social media was in its infancy [6]. In the majority of the current research, there is a view that digital technology, particularly social media, has amplified the problem of health misinformation. The risk most frequently identified, either explicitly or implicitly, is the susceptibility of ordinary users, who may be lacking sophisticated levels of health and digital literacies, to health misinformation. Further risks noted in the literature include disinformation disseminated by organized trolling networks and bots that can be hard to distinguish from human users. The recognition of these risks underpins an emerging policy discourse about the threat of health misinformation, particularly the role of social media in its spread, in which health information and misinformation has become a politicized issue. From one policy perspective, there is an assumption that social media users are vulnerable, even passive, recipients of health misinformation rather than reflective interpreters of the available information. The corollary of this is that correcting misinformation with authoritative knowledge will solve the problem. However, a survey of the literature suggested that neither assumption fully expresses the complexity of how health misinformation is disseminated, received, and used via the internet. This may be because although there is a growing body of evidence on the extent of online health misinformation, there is much less research about what users do with health misinformation, why users consume health misinformation, and why (and which) people believe health misinformation [7-9].

## Methods

### Overview

In this scoping review, we reviewed the current state of knowledge regarding the prevalence of online misinformation before and during the COVID-19 pandemic and the impact that has on users’ understanding of health information. We considered this context with special reference to patients’ understanding, health practitioners’ practice in response to that, and policy makers’ concerns. The pressures and distractions that health professionals face in attempting to mitigate the impacts of online health misinformation are discussed in relation to patients’ health and digital literacies and the politicization of health information and misinformation.

### Information Sources

We conducted a comprehensive literature search to identify relevant studies that explored health misinformation on social media platforms. The search was conducted across multiple electronic databases, including PubMed, Scopus, Web of Science, and Google Scholar.

### Search

The search terms included a combination of relevant keywords and phrases, including “health misinformation,” “social media,” “online health communities,” and “COVID-19 pandemic.” The search was not limited by publication date. Detailed search strategies are provided in Multimedia Appendix 1.

### Study Selection

Our study selection process followed a scoping approach, where we aimed to identify and include studies that provided insights into the prevalence and challenges of health misinformation on social media platforms. Initially, 2 researchers independently screened titles and abstracts of the identified articles to determine their relevance. Articles that did not meet the inclusion criteria were excluded at this stage.

### Inclusion Criteria

Articles were included if they discussed health misinformation on social media, addressed the challenges posed by health misinformation, or were relevant to the period before, during, and after the COVID-19 pandemic.

Any disagreements between the 2 researchers were resolved through discussion and consensus. Full-text articles were then retrieved for the remaining studies, and a further assessment of eligibility was conducted based on the same inclusion criteria.

### Data Extraction

We gathered information on (1) study objectives, (2) research methods, (3) findings, and (4) key themes related to health misinformation. This process was performed independently by 2 researchers, and any discrepancies were resolved through discussion.

### Data Synthesis and Analysis

We adopted a scoping review content analysis approach to analyze the data extracted from the selected articles. The analysis process involved identifying key themes and patterns related to health misinformation on social media. The content analysis allowed us to gain a deeper understanding of the challenges posed by health misinformation and the strategies for its mitigation, both before and during the COVID-19 pandemic.

## Results

### Results of Search

**Figure 1:**
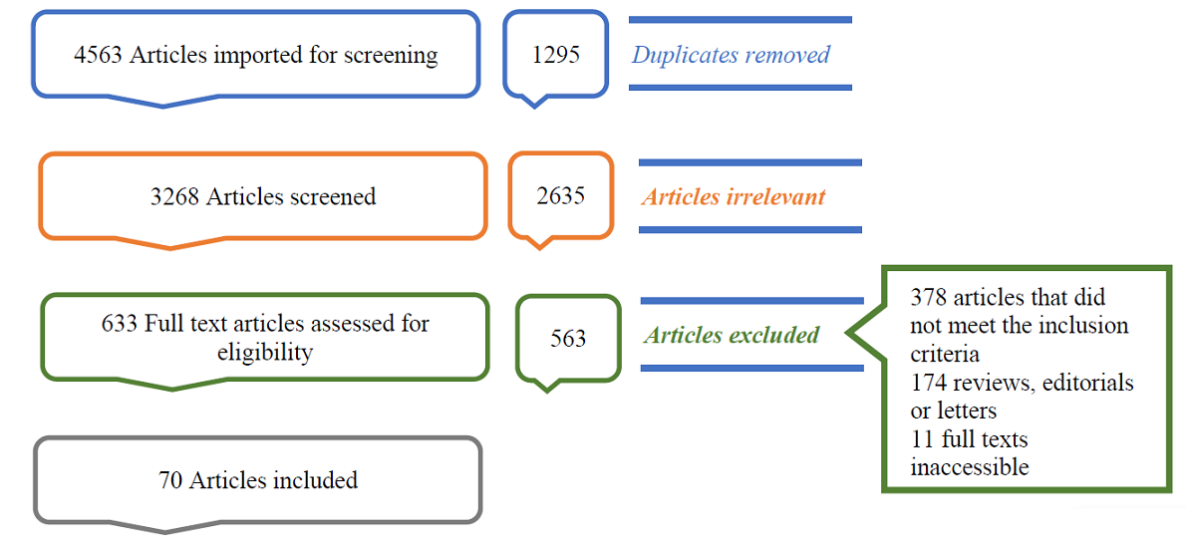
PRISMA (Preferred Reporting Items for Systematic Reviews and Meta-Analyses) flowchart.

In our article selection process (Figure 1), we initiated our search by identifying a total of 4563 articles from various databases. Following the removal of duplicates, 1295 articles were excluded, leaving us with 3268 unique articles. Subsequently, these articles underwent an initial screening, which involved evaluating their abstracts and titles, resulting in the exclusion of 2635 articles that did not align with our inclusion criteria. Further scrutiny was applied during full-text screening, which was conducted on 633 articles. Among these, 563 articles were found ineligible due to reasons such as not meeting the inclusion criteria (n=378 articles), being classified as literature reviews, editorials, or letters (n=174 articles), or the full texts being inaccessible (n=11 articles). A total of 70 articles were ultimately included in this scoping review.

### Characteristics of Included Documents (n=70)

The majority (65/70, 93%) of documents were published in the last 10 years and originated predominantly in North America (42/70, 60%), Europe (19/70, 27%), and Asia (11/70, 16%). The funding sources were mainly public (61/70, 87%). The documents were classified as original research papers (38/70, 54%), subjective “opinion” articles (editorials, viewpoints, commentaries, and letters to the journal; 11/70, 16%), and knowledge syntheses or reviews (9/70, 13%) which included systematic reviews (n=6), descriptive reviews (n=2), and 1 integrated theoretic review.

### Extent and Impact of Health Misinformation Disseminated Across a Range of Outlets

#### Overview

This section will review the literature concerning the extent and impact of the problem of health misinformation, including the spread of antivaccination discourse. In a study by Wood et al [10] among health practitioners in North Carolina, 94.2% of the respondents reported encounters with patient health misinformation within the previous year. While the sources of this misinformation were not broken down and identified by Wood et al [10], several other studies linked patient health misinformation to the prevalence of health misinformation on social media sites, identifying the latter as a significant problem [4,11-15]. There is a growing consensus among researchers, health professionals, and policy makers about the need to confront, challenge, and even prevent the online dissemination of health misinformation [16]. Since the emergence of online social networks, users have increasingly sought and shared health information on social media sites. It is estimated that around 70% of adult internet users search health matters on the web. With the emergence of social media platforms, there has been a rise in “peer-to-peer health care,” through which individuals seek and share health information, forming online health communities with others who have similar health concerns [3]. In addition, health organizations and health professionals are increasingly using social media to disseminate and promote health information and advice. The opportunities for sharing and promoting good health information via the internet are evident, and it is important to acknowledge that in online health communities, users share experiences and receive and give different kinds of support, including emotional support, to cope with specific health conditions. However, the medium has also enabled the dissemination of health misinformation, and the prosocial aspects of sharing are also likely to involve the sharing of misinformation, putting the health of users at risk [3].

#### Misinformation Spreads on Social Media

There is a high degree of consensus among researchers that mainly because of the increasing popularity of social media, the internet has become a space for the dissemination and amplification of “fake news,” misleading information, and rumor, including health misinformation and antivaccine conspiracy theories [17]. The COVID-19 pandemic has heightened these concerns, resulting in a proliferation of recent studies and rapid reviews focusing on the online spread of misinformation. Lee et al [18] proposed that the proliferation of health misinformation during the COVID-19 pandemic became a major public health issue. At the earliest signs of the emerging COVID-19 pandemic, the director-general of the World Health Organization, Tedros Adhanom Ghebreyesus, speaking at the February 2020 Munich Security Conference, expressed concern about the risk of an infodemic of health misinformation disseminated via social media, identifying “vaccine hesitancy” as 1 of the top 10 global health threats [19]. Bapaye and Bapaye [20] agreed that the risks of misinformation on social networking sites constitute a global issue, referring specifically to the COVID-19 infodemic.

However, this is not in itself a new problem; longstanding concerns about “fake news” and misinformation in traditional media have been evident since the early decades of the 20th century [21], and the prevalence of misinformation on internet platforms certainly predates the COVID-19 pandemic. Therefore, because the COVID-19 pandemic has only intensified the concern regarding health misinformation, it might be more appropriate to see the pandemic as symptomatic of, and crystallizing, the challenges of countering health misinformation in the digital age, as the development of digital technology and the internet have brought about profound changes in the capacity of both misinformation and disinformation to spread globally and amplify rapidly [4].

Suarez-Lledo and Alvarez-Galvez [16] undertook a review of 69 studies of health misinformation on social media to identify the main health misinformation topics and their frequency on different social media platforms. The studies surveyed used a variety of research methods, including social network analysis (28%), evaluation of content (26%), evaluation of quality (24%), content/text analysis (16%), and sentiment analysis (6%). Suarez-Lledo and Alvarez-Galvez [16] concluded that the incidence of health misinformation was highest on Twitter, in particular, regarding the use of tobacco and other drugs, with some studies citing 87% of such posts containing misinformation. However, health misinformation about vaccines was also prevalent, with around 43% of posts containing misinformation, with the human papillomavirus vaccine being the most affected. This review by Suarez-Lledo and Alvarez-Galvez [16] confirmed many of the findings from earlier surveys. For example, in their survey of 57 articles, Wang et al [4] found that the most frequently discussed topics were regarding vaccination and infectious diseases, including Ebola and the Zika virus. Other topics such as nutrition, cancer, water fluoridation, and smoking were also prevalent. The studies they surveyed had tended to find that a high degree of misinformation on these topics was being shared and liked on social media.

Lee et al [18] conducted a cross-sectional online survey in South Korea to examine the prevalence of COVID-19 misinformation and the impact of exposure to COVID-19 misinformation on beliefs and behaviors. They found that exposure to COVID-19 misinformation was associated with misinformation belief, which then resulted in fewer preventive behaviors. Therefore, they highlighted the potential of misinformation to undermine global efforts in disease control and argued that public health strategies are needed to combat the proliferation of misinformation. Bapaye and Bapaye [20] conducted a cross-sectional online questionnaire survey of 1137 WhatsApp users in India. They noted that most research on the prevalence of misinformation in social media has focused on Twitter and Facebook and on the Global North. Measured by age, researchers found that users aged >65 years were the most vulnerable to accepting the veracity of messages containing health misinformation (K=0.38, 95% CI 0.341-0.419) Respondents aged 19 to 25 years displayed much lower vulnerability (K=0.31, 95% CI 0.301-0.319) than those aged >25 years (*P*<.05). Measured by occupational category, users employed in nonprofessional occupations had the highest vulnerability (K=0.38, 95% CI 0.356-0.404); this was significantly higher than those of professionals and students (*P*<.05). Notably, the vulnerability of health professionals was not significantly different from those of other occupation groups (*P*>.05).

The authors concluded that in a developing country, WhatsApp users aged >65 years and those involved in nonprofessional occupations are the most vulnerable to false information disseminated via WhatsApp. Crucially, they noted that health care workers, who might be expected by laypersons to have expert knowledge, were as likely to be vulnerable to health misinformation as other occupation groups.

#### Antivaxxer Spread Before, During, and Beyond the COVID-19 Pandemic

Much of the current unease from researchers, understandably, centers on health misinformation about vaccines in the wake of the COVID-19 pandemic. In particular, there is concern about the growth and spread of so-called antivaxxer misinformation and beliefs. In 2019, the United States had its biggest measles outbreak in 30 years, with most cases involving people who had not been vaccinated. Hotez [22] claimed that much of the reason for the growth of antivaccine beliefs is because of a campaign of misinformation. He argued that social media sites are meeting places for the sharing of antivaccine views. To evade social media platforms’ automated moderation tools, which tend to focus on words, several antivaxxer groups, including one with around 250,000 members, began using visual codes, such as the carrot emoji, to hide antivaxxer content.

However, some of the misinformation has gained credibility because it has come from sources that laypersons would expect to be trustworthy. For example, in 1998, the British medical journal *The Lancet* published a paper by Dr Andrew Wakefield claiming a link between the measles, mumps, rubella vaccine and the onset of autism spectrum disorder. Wakefield’s paper was later rebutted, and an overwhelming body of evidence now refutes its conclusions [23]. However, despite long being discredited, Wakefield’s claims have remained a part of the antivaccine discourse. The persistence of the antivaccination narrative demonstrates the power of such discourses even in the face of evidence to challenge them.

Although strong antivaccine beliefs, and the more ambivalent attitude of vaccine hesitancy, have been around as long as there have been vaccines, until recent decades, they were on the margins. However, evidence supports the claim that they have been gaining momentum in the United States and Europe.

A survey by Skafle et al [24] aimed to synthesize the results from 19 studies in which the effect of social media misinformation on vaccine hesitancy was measured or discussed. The authors noted that the “vast majority” of studies were from industrialized Western countries. Only 1 study contained misinformation about autism as a side effect of COVID-19 vaccines. Nevertheless, the studies implied that information spread on social media had a negative effect on vaccine hesitancy and uptake. The conclusions from Skafle et al [24] were supported by data from online polling agencies. For example, a US YouGov poll from May 2020 found that only 55% of respondents would definitely take a COVID-19 vaccine if one were to become available, whereas 19% of respondents said that they would refuse and 26% were still undecided [25].

While much of the research about online vaccine discourse comes from the United States, there is also evidence that vaccine hesitancy has risen elsewhere. For example, in an Ipsos-MORI survey taken in December 2020, only 40% of respondents in France said they would take a COVID-19 vaccine, a figure symptomatic of a steep and swift decline in vaccine confidence in France [26]. However, interestingly, the same Ipsos-MORI poll indicated a rise in vaccine confidence among respondents in the United States since the earlier YouGov poll, cited earlier, by approximately 10% to 65%, and respondents in the United Kingdom expressed a still higher willingness to take a COVID-19 vaccine at approximately 77%. It is notable that in the United States and United Kingdom, the Ipsos-MORI results came after a period of intermittent lockdowns. The contrast with the results from France is, nevertheless, striking.

### Understanding the Challenges Surrounding Health Misinformation

Here, we consider the challenges created by health misinformation on the web: (1) the role played by malicious actors on social media in spreading vaccine disinformation and misinformation and (2) how contextual and cultural issues have different effects on patients’ understanding of what is considered genuine, valid, and authentic health information.

#### Spread of Health Misinformation on Social Media by Malicious Actors

One strand of research presents the issue of health misinformation as a contest between trolls and bots on the one hand and the voices of trustworthy public health agencies on the other [6]. This view was supported by Hotez [22] and Broniatowski et al [11]. The latter investigated the role of bots and trolls as malicious actors mobilizing vaccination discourse on the web. Their study focused specifically on vaccine-related health messaging on Twitter. Comparing the rates of vaccine-related messages, they found that sophisticated bots and Russian trolls tweeted at higher rates than “average users.” However, the respective content from bots and trolls differed. Whereas bots communicated antivaccine messages, Russian troll accounts provided a seemingly balanced discussion of both provaccination and antivaccination arguments, implying an equivalence between them. The authors argued that amplifying and normalizing a debate is done with the purpose of sowing discord and may lead to undermining public confidence in scientific consensus about the effectiveness of vaccines. Wang et al [4] acknowledged that it is a challenge to readily distinguish between misinformation and disinformation on the web. They noted that disinformation, such as antivaccine propaganda, can unknowingly be spread by users with genuine concerns [4], as individuals increasingly seek health and healthy lifestyle information via the internet.

#### Contextual Factors Influencing the Reception of and Responses to Misinformation: Politicization of the Problem of Health Misinformation

The identification of online trolls, bots, and orchestrated networks as major contributors to the spread of health disinformation and misinformation is now part of mainstream political discourse in the United States. On July 16, 2021, a quarrel broke out between the president of the United States, Joe Biden, and Facebook over the spread of health misinformation on the company’s social media platforms. Speaking to journalists, Biden blamed social media companies for a rise in the number of deaths from COVID-19 among the unvaccinated in the United States. Referring explicitly to Facebook, the president claimed that by allowing the proliferation of health misinformation on its platforms, the company was “killing people” [27]. Discursive interventions from politicians are never neutral; nevertheless, Biden’s claim about the impact of health misinformation on social media is backed up by many of the studies surveyed for this paper. Facebook immediately rebutted Biden’s accusation by citing their rules, introduced in February 2021, which banned posts that make identifiably false claims about vaccines. Furthermore, Facebook challenged Biden’s claim by asserting that not only has Facebook provided more authoritative information about COVID-19 and vaccines than any other internet site, reaching 2 billion people with such posts, but also that the platform’s vaccine finder tool had been used by more than 3 million Americans.

These figures suggest that although antivaxxer groups find ways to evade detection, their reach may be countered by that of information grounded in current science. A spokesperson for the company said that, far from killing people, “The facts show that Facebook is helping save lives. Period” [27]. The argument between Biden and Facebook may indeed signal more lay awareness of the problem and echo the concerns of the recent academic research about the dissemination of health misinformation by organized bot and troll networks. Framed as it is, in terms of apportioning the blame for the spread of health misinformation, Biden’s intervention mirrors much of the academic discourse in the United States on the subject. However, it is also symptomatic of the politicization of health misinformation, arguably accelerated by the COVID-19 pandemic, which may thwart evidence-based decision-making. This point was emphasized strongly by Kyabaggu et al [5]. They framed the problem of pervasive misinformation and disinformation in terms of prime movers and beneficiaries who use it to advance sociopolitical agendas and entrench asymmetrical power, especially in times of uncertainty and threat, such as the COVID-19 pandemic.

Kyabaggu et al [5] identified government failures to adopt evidence-informed decision-making. They noted that such failures have costs that not only are economic but, crucially, result in poorer health outcomes. They cited as an example the United Kingdom government’s initial prevaccine herd immunity strategy. The intention of this strategy was to allow SARS-CoV-2 to indiscriminately spread to a critical mass to build up population immunity. The authors noted that this was “a particularly concerning example of evidence framing by a government.” Kyabaggu et al [5] argued that public acceptance of health risk messages and adoption of health-protecting behaviors is highly contingent on the degree to which governments engage in evidence-informed decision-making and communicate this basis effectively. The authors cited several instances of government actors failing to recognize misinformation, disseminating inconsistent or inaccurate information, and not using evidence- and information-based decision-making processes. In recent years, the public policy discourse in the United Kingdom has been veering away from evidence- and information-based decision-making, as politicians have denounced “experts” and their “influence” on policy [28,29].

Finally, Gruzd et al [30] reported on the impact of coordinated link-sharing behavior to spread and amplify conspiracy-related misinformation. They found a coalition of Facebook accounts that engaged in coordinated link sharing behavior to promote COVID-19 related misinformation. This coalition included US-based pro-Trump, QAnon, and antivaccination accounts.

#### Contextual Factors Influencing the Reception of and Responses to Misinformation: Health Literacies and Inequality

While the approach of Broniatowski et al [11], for example, provided a persuasive account of ways in which online health misinformation can be disseminated, there are limitations to this approach, as it did not provide an account of how users respond to the misinformation they encounter. The responses of ordinary users were assumed rather than investigated. Research by Vosoughi et al [31] provided a caveat to the claim that it is bots that accelerate the spread of misinformation. Their work supported that of Broniatowski et al [11] in suggesting that bots spread accurate and false information at the same rate. However, Vosoughi et al [31] also explained that misinformation spreads more rapidly than accurate information because humans, rather than bots, are more likely to spread misinformation [31]. This claim was further supported by Wang [32], who suggested that in democracies, where ideas compete for attention in a marketplace, accurate scientific information, which, for the layperson, may be boring or difficult to understand, is easily crowded out by information that is more easily grasped or sensational. Mokhtari and Mirzaei [12] located this problem specifically in the context of the COVID-19 pandemic. They considered that high mortality from COVID-19, its complexity, and its unknown features resulted in fear, anxiety, and mental pressure among people worldwide. To allay anxiety, people needed health information literacy, defined by the American Library Association as a set of abilities individuals require to recognize when information is needed and to locate, evaluate, and use it effectively [33]. In addition, Wang [32] noted that individuals are differentially vulnerable to health misinformation depending on their level of health literacy and that models need to account for this. Mokhtari and Mirzaei [12] argued that not only information and health literacies but also media literacy are needed. However, studies in the field of health literacy suggest that significant inequalities in health and digital literacies exist.

Researchers have argued that “vastly undervalued and unrecognized” health literacy ought to be considered the best “social vaccine” for preventing COVID-19 in populations [5]. However, inequalities in health literacy persist. Kyabaggu et al [5] defined health literacy as encompassing cognitive and social skills that determine individuals’ motivation and ability to access, understand, and use information, including quantitative health risk information, in ways that promote and maintain good health across the life course. They asserted that health literacy is an essential self-management skill and community resource for health, noting that health literacy is positively associated with patients’ involvement in clinical decision-making, willingness to express health concerns, and compliance with clinical guidance. However, despite research demonstrating the importance of health literacy, evidence, even from high-income countries, suggested relatively low levels of health literacy.

Kyabaggu et al [5] drew a link between health literacy and digital literacy. They suggested that the latter can be understood as health literacy in digital information and technology spaces. They argued that inequalities in health outcomes are exacerbated by a widening digital divide. While digital technology in health and social contexts presents both new risks and opportunities for equity in different information audiences, the ways in which power and privilege operated in the COVID-19 misinformation discourse have not been sufficiently examined. Although socially and economically disadvantaged groups were at a greater risk of exposure to COVID-19, their voices and experiences were often marginalized. In addition, inequalities in access to accurate information are not only related to issues of digital access and literacy but are also situational. For example, disadvantaged individuals may have fewer social connections, and low pay may necessitate longer working hours, militating against individuals having the resources of time and energy to seek out accurate health information and enhance their level of health literacy.

The experiences of specific groups may also go unreported. Quraishi [34] addressed the impact of misinformation on South Asian students—a fast-growing group in the United States, but one that often receives little media attention. Quraishi [34] concluded that there is a relationship between the COVID-19 pandemic and students’ academic performance and mental health, as well as an increase in the spread of misinformation regarding COVID-19 public safety guidelines.

Older adults can be a vulnerable group in relation to their comparatively poor digital literacy. Zhou et al [35] reported on the accuracy of older adults in judging health information credibility. They found that on average, participants only successfully judged 41.38% of health articles. Attractive headlines increased participant credibility judgments on the content, and of the articles shared with others, 62.5% contained falsehoods.

#### Contextual Factors Influencing the Reception of and Responses to Misinformation: Cultures and Values

Larson and Broniatowski [19] argued that developing the kinds of literacy advocated by Mokhtari and Mirzaei [12] and Tully et al [2] will not address the deep-seated problems they identified. The work by Kyabaggu et al [5] supported this, and noted that the infodemic crisis is not merely a health and digital literacy issue. Some demographics may be more vulnerable to persuasive communication from broader sociocultural forces. Kyabaggu et al [5] argued that in considering the social determinants of health, attention must be paid not only to digital and health literacies but also to the ways in which these literacies coexist and interact with other influences. Larson and Broniatowski [19] suggested that one of the strongest determinants of vaccine confidence or vaccine hesitancy is the level of trust or distrust in the institutions that produce vaccines. A higher level of trust encourages the willingness to accept a high level of risk for a greater benefit. A lower level of trust militates against the acceptance of even a low level of perceived risk. For Larson and Broniatowski [19], it is not simply the presence of misinformation on social media networks but the social and cultural context of users’ reception of that information that influences responses. Health information operates in a complex and contentious social world. Individuals and communities respond to new information in terms of already developed political, cultural, and social values that influence whether they trust or distrust authority. Populations may be characterized by trust or mistrust of scientific institutions and government. Trust has been eroded through the exposure of fraud, research scandals, and misconduct by major multinational pharmaceutical companies, for example. Communities may be predisposed to distrust the government and its agents depending on their own status or identity. According to Goldenberg [36], these contexts can make misinformation and health conspiracy theories compelling.

### Strategies to Correct Online Misinformation

We address the additional pressures on health professionals in communicating accurate information to mitigate the effects of misinformation, particularly with regard to the additional requirements imposed as a result of the precautions being taken during the pandemic. One area of disagreement in the literature concerns the usefulness of user correction response.

#### Research Into User Correction Strategies

There is some disagreement as to whether engagement with misinformation by users spreads and reinforces it or even whether extended debates over health misinformation cause users to doubt the possibility of knowable facts. For example, Broniatowski et al [11] argued that when ordinary users directly confront vaccine-skeptic messages from bots, it only serves to legitimize the “debate.” By contrast, Tully et al [2] argued that social media users have a role to play in either spreading or stopping the spread of misinformation across platforms. Their research aimed to uncover what factors influenced users’ responses. Tully et al [2] acknowledged that a range of factors can influence the spread or prevention of misinformation, including the behavior of malicious actors such as bots and trolls; the platform’s terms of service; and content moderation policies. As already noted, while most users are not creators of misinformation, they may spread and amplify it by liking, sharing, or replying. In opposition to the work of Broniatowski et al [11], Tully et al [2] argued that the content of engagement is particularly important, as their research suggested that multiple corrections by social media users may be required to reduce misperceptions. However, they claimed that most people simply ignore misinformation when they see it on social media.

Tully et al [2] noted the promise in mobilizing users to engage in such correction, given the vast numbers of users on these sites, in comparison with professional fact-checkers and health authorities.

They considered whether the tone of a correction would influence perceptions of the credibility of the message. However, despite some mixed evidence, they concluded that overall, the tone was not a significant factor and that neutral, affirmative, and uncivil corrections were all effective at reducing misperceptions. They found that participants were generally unlikely to reply to the misinformation tweet. However, their content analysis of hypothetical replies suggested that when users did reply, they mainly provided correct information, particularly after seeing other corrections. Tully et al [2] concluded that user corrections offer “untapped potential” in responding to misinformation on social media, but further work is needed to consider how users can be mobilized to provide corrections, given their overall unwillingness to reply. However, a limitation of the experimental approach of Tully et al [2], acknowledged by the researchers, is that in asking individuals what they would hypothetically do, this may not reflect what they actually do in a real social media setting, especially in relation to an issue they care more strongly about. Although the experiment gauged attitudes, it did not delve into how strongly these attitudes were held. It is also not clear to what degree corrections were effective at reducing misperceptions and how reductions were measured.

By contrast, the results of experimental studies by Ittefaq [37] and Mourali and Drake [38] suggested that correcting misinformation is by no means a straightforward proposition. They noted the previous research on rebuttal, which suggested that properly designed corrections can mitigate the effects of misinformation. However, such studies have tended to compare responses to misinformation followed by correction with responses of a control group that receives no correction or receives an alternative correction. Mourali and Drake [38] argued that this static approach misses the dynamic nature of social media debate. They noted that the correction of misinformation is generally followed up with a rebuke by the original poster, inciting further correction and prolonged back-and-forth debate. Mourali and Drake [38] cited previous studies showing that exposure to conflicting information about health topics, including mammography, nutrition, and the human papillomavirus vaccine, may increase confusion and negative attitudes toward that particular health topic. The researchers found that initial exposure to misinformation had a negative impact on attitudes and intentions toward masking, consistent with previous studies that concluded that exposure to misinformation negatively impacts attitudes and intentions toward behaviors favored by science. Also consistent with previous research, they found that the first correction of the false claim improved attitudes and intentions toward masking. The authors suggested that this effect is partially explained by a decrease in the perceived strength of the argument underlying the false claim. However, this initial improvement diminished on further exposure to false claims and refutation attempts. This finding confirmed their hypothesis that extended exposure to false claims and refutation attempts appears to weaken belief in the possibility of objective knowledge, leading to less positive reactions toward masking as a science-based behavior. They concluded that the level of exposure to contradictory information needs to reach a certain threshold before it affects perceived truth objectivity. However, although people are more likely to share misinformation when its content is consistent with their existing beliefs or when its message is simple, direct, or sensational, correcting misinformation does reduce its likelihood of being shared on social media, an effect that persists even after multiple exposures.

Mourali and Drake [38] noted that each social media platform exhibits particular interaction norms, which may impact how users interpret the conversation. As their study was limited to a single platform, Reddit, and the debate was restricted to 4 exchanges between only 2 protagonists, the researchers acknowledged that these aspects limit the generalizability of the results. They suggested that future research could attempt to replicate their findings on different social media platforms, and to include more than 2 protagonists and more than 4 exchanges. They noted further that although extended debates are common on social media, it is not known how frequently they occur, echoing the comments by Suarez-Lledo and Alvarez-Galvez [16] that the extent of misinformation is not clear.

In contrast to the fairly sanguine view of Tully et al [2] about the potential of users to spread corrective information, Mourali and Drake [38] problematized the position, pointing to the potential for more complex and uncertain outcomes, whereas Larson and Broniatowski [19] argued that although the importance of correcting misinformation, item by item, should not be diminished, only if underlying issues driving misinformation are addressed can, for example, long-term vaccine confidence in populations be sustained. They argue that simply responding to misinformation with factual corrections is not likely to reverse the dissent that has been evident among antivaxxers or to necessarily persuade the more ambivalent vaccine-hesitant individuals. They identified deeper social and cultural issues at play, which have been discussed in this paper in the previous sections.

#### Research Into Effective Models to Accomplish the Automatic Detection of Health Misinformation in Online Health Communities

Here, we consider examples of research into the automatic detection of health misinformation in online health communities. Zhao et al [3] began from the premise that there is a vast amount of health misinformation, creating a challenge for health communities in identifying misinformation. Rather than relying on users’ ability to correct misinformation, they proposed that there is a need for an effective model to achieve automatic detection of health misinformation in online health communities. This view was also put forward by Weinzierl and Harabagiu [39]. Focusing specifically on COVID-19 vaccine misinformation, they argued that automatic detection of misinformation on social media is an essential first step in delivering interventions designed to address vaccine hesitancy.

Zhao et al [3] identified much of the existing analysis as concentrating on the linguistic features of communications only. They wanted to examine the underresearched area of whether integrating user behavioral features with linguistic features, sentiment features, and topic features could effectively distinguish misinformation from accurate information in online health communities. Their study combined the aforementioned features to build a detection model targeting misinformation in online health communities’ contexts. The behavioral features targeted were discussion initiation, interaction engagement, influential scope, relational mediation, and informational independence. Descriptions of these behavioral features are reproduced in Table 1.

**Table 1:** Descriptions of the behavioral features.

Behavioral feature	Measurement	Description
Discussion initiation	The number of threads a user created	To reflect the activity of a user in terms of initiating new discussions
Interaction engagement	The number of replies and the number of replies to a reply a user created	To reflect the activity of a user in terms of interacting with other users
Influential scope	Degree centrality	To reflect the potential communication ability of a user
Relational mediation	Betweenness centrality	To assess the potential of a user for the control of communication in the community
Informational independence	Closeness centrality	To assess the ability of a user to instantly communicate with others without going through many intermediaries

The authors tested their detection model on a data set collected from a real online health community, selecting as their data source Zibizheng Ba, an autism forum on the Baidu Tieba online health community site hosted by the Chinese web service Baidu. Baidu Tieba claims to be one of the largest interest-based discussion platforms in China. Users can generate topic-based discussion forums on the platform, share information, and make friends with other users. Posts on Baidu Tieba are indexed by Baidu, China’s most popular search engine, so users can readily find misinformation when searching for health-related information through the search engine. The authors developed a python-based web crawler to collect data from the forum. To train the health misinformation detection model, 5000 records were sampled from the whole data set by stratification according to 3 types of records (ie, thread, reply, and reply to reply) using stratified sampling methods. Therefore, the constituent types of the records (ie, thread, reply, and reply to reply) in the sample data set were consistent with the composition of the whole data set.

The researchers applied the elaboration likelihood model (ELM). The model, originally developed by Petty and Cacioppo [40] to explain attitude change, has been used extensively in advertising to try to influence consumers.

Overall, 4 types of misinformation were identified through their coding analysis, and the model correctly detected about 85% of the health misinformation. Their results also indicated that behavioral features were more informative than linguistic features in detecting misinformation. The authors concluded that their results not only demonstrated the efficacy of behavioral features in health misinformation detection but also offered both methodological and theoretical contributions to misinformation detection by integrating the features of messages as well as the features of message creators. Others have also highlighted the problems posed by misleading visual information [41].

It is worth noting that during the pandemic, the UK National Health Service (NHS) began using Twitter to promote provaccine messaging, which closely follows a combination of the features suggested by Zhao et al [3]. When users searched for the term “vaccine” or related terms, the top post was a message prominently displaying the NHS logo, identifying it as reputable and trustworthy. The tweets contained links to NHS websites providing information about vaccines and COVID-19. The posts differed in linguistic content and visual design. For example, one featured only written text on a white background and stated in bold, “Know the facts.” Another featured a large image of a happy minority ethnic family, washing dishes together, with the message that the COVID-19 vaccine decreases household transmission by up to half. The contrasting designs suggest that the message was targeted specifically to users’ timelines. It was also apparent that elements of ELM were being applied, combining the features identified by Zhao et al [3] in different ways.

Weinzierl and Harabagiu [39] adopted a different method than Zhao et al [3], reversing the more commonly used classification approach. The authors of each study claimed strong results in identifying health misinformation on social media platforms. However, Nabożny et al [42] argued that the current automatic systems for assessing the credibility of health information are not sufficiently precise to be used without supervision by human medical expert annotators.

Barve and Saini [43] have reported on their use of automated fact-checking using a coded content similarity measure (CSM). In this approach, the CSM showed improved accuracy (91.06%) compared to the accuracy of the Jaccard similarity measure (74.26%). Further, the algorithmic approach outperformed the feature-based method.

Neither Zhao et al [3] nor Weinzierl and Harabagiu [39] recorded what happens when misinformation is detected. Research from Broniatowksi et al [44] suggested that once detected, steps taken by social media platforms such as content removal or deplatforming may not be effective in stemming the spread of misinformation and may even be counterproductive. Social media platforms use a combination of “hard” and “soft” content remedies to reduce the spread of health misinformation. Soft remedies include warning labels attached to content and downranking of some content in web searches, whereas hard remedies include content removal and deplatforming of accounts. Hard remedies are controversial and have given rise to accusations of censorship. For the authors, short-term evidence for the effectiveness of hard remedies is in any case mixed, and long-term evidence is yet to be examined. Their study focused on Facebook and found that while hard remedies did reduce the number of antivaccine posts, they also produced unintended consequences. Provaccine content was removed, and engagement with the remaining antivaccine content repeatedly recovered to prepolicy levels. Worryingly, this content became more misinformative, more politically polarized, and more likely to be seen in users’ news feeds. The authors explain these results as a product of Facebook’s architecture, which is designed to promote community formation. Members of communities dedicated to vaccine refusal seek out misinformation. To meet this demand, and to circumvent content moderation efforts, antivaccine content producers post links to external sources of misinformative content, such as Bitchute, Rumble, Gab, and Telegram, in lieu of more mainstream platforms that had implemented similar content removal policies (eg, YouTube and Twitter). Broniatowski et al [44] argued that Facebook’s policy reduced the number of posts in antivaccine venues but was not successful in inducing a sustained reduction in engagement with antivaccine content, including misinformation. The authors noted that alternative platforms often host politically extreme right-wing content. Therefore, they argued that Facebook’s content removal policies may have the unintended consequence of radicalizing their audiences, and their findings suggested the need to address how social media platform architecture enables community formation and mobilization around misinformative topics when managing the spread of online content.

These studies advocate for the automatic detection of health misinformation. However, work that calls into question the ability of automatic detection to operate without human intervention has also been discussed. In addition, there are questions raised in the literature about what should be done when misinformation is detected and concerns about whether content removal or deplatforming of accounts are the most effective ways to reduce the spread of health misinformation or may even be counterproductive.

### The Roles of Health Practitioners

#### Overview

The discussion so far has highlighted the complex and multifaceted dimensions of the context of online health misinformation in which health practitioners must operate. As noted in our introduction, a study of health practitioners in North Carolina found that nearly 95% had encountered patient health misinformation within the previous year [10]. There is very little research on the amount or effectiveness of training received by health professionals to prepare them for engaging with patients about health misinformation. Wood et al [10] found that most respondents had not received relevant training despite overwhelmingly reporting encountering health misinformation.

Nevertheless, within the literature, there is no shortage of advice from researchers and health professionals addressed to health practitioners on how to approach and correct health misinformation. This advice stems from both original research studies and reviews of best practices featured in peer-reviewed medical and health journals. Such advice centers on the need for health practitioners to understand misinformation and how to address it. Health practitioners are advised of the need to be aware of health myths and urged to dismantle them in providing accurate health guidance [45,46]. Practitioners are further advised that misinformation and pseudoscience are appealing to those seeking certainty because they present information in absolutes, whereas medical science is often ambiguous and contingent. Health practitioners are also encouraged to learn how to message more clearly and to mimic the strategies of misinformation [45]. One study recommends that “practitioners familiarize themselves with the tools of scientific enquiry and consider the pros and cons of various conspiracy evaluation guidelines” [47]. Thompson [48] reports on the activity of health professional influencers and pedagogues in combating misinformation. However, the effectiveness of such social media influencers who are also health professionals remains unclear. At the same time, there is some acknowledgment in this body of literature that misinformation cannot simply be offset with facts, confirming the challenges, discussed earlier, of simply engaging in online refutation. Addressing misinformation also depends on meeting patients’ emotional needs [45,49].

In this context, the one-to-one patient-provider relationship in the practice setting is perceived as paramount [45]. As suggested by much of the research, source credibility, or trust, is understood to be the strongest driver of effective correction strategies [50]. It is argued that health care practitioners have the unique opportunity to guide patients toward high-quality, evidence-based medical information [10]. However, it is also noted that practitioners will need patience in their efforts to persuade patients to abandon strongly held self-beliefs, however harmful. Doing so may mean patients relinquishing membership of online communities that have become integral in their lives and even their identities. As noted earlier, belief in misinformation is often persistent in the face of evidence. Success is more likely when individuals are encouraged to reexamine their information sources, alongside new information providing additional context, rather than simply characterizing the individual’s beliefs as wrong [51]. Kyabaggu et al [5] commented that good health communication needs to be tailored to the underlying cause of the misinformation problem, and efforts should be made to take on board inequalities within populations to create accurate, low-barrier, targeted health risk messaging. Skafle et al [24] contended that to challenge misconceptions, false claims need to be openly addressed and discussed with both cultural and religious awareness in mind. Guidance for practitioners noted that while responding to patient questions about alternative or unproven therapies may become laborious, a strong bond of trust between health practitioner and patient gives a patient a feeling of being supported and increases their adherence to treatment [52]. Rather than waiting for patients to raise misinformation issues, health care practitioners are advised to anticipate and proactively address potential misinformation and myths with patients. For example, the mortality rate for pediatric cancer has risen during the COVID-19 pandemic because of delayed access to medical care, but misinformation related to COVID-19 may also be a contributing factor [53]. The literature highlights the challenge of navigating the information and misinformation and the need for health practitioners to communicate with their patients more effectively. However, such efforts are not always successful. Some of the factors that may prevent effective communication of good health information have already been raised in this paper. They are revisited and discussed in the next section, along with other stressors for health practitioners.

#### Stressors for Health Practitioners

Challenges for health practitioners include time pressures and the additional burdens placed on them during the COVID-19 pandemic. These additional pressures add to the issues health practitioners face in trying to mitigate the impact of misinformation. The following is a brief overview of these issues.

On the one hand, administrative burdens placed on practitioners frequently deny them time for dialogue with their patients [52]. On the other, in different contexts, practitioners may be coping with a lack of proper facilities; poor infrastructure for patient care; insufficient or ineffective personal protective equipment; lack of awareness among the general population; poor compliance with preventive methods; and the fear of being infected with the virus, as they too are exposed to misinformation. During the COVID-19 pandemic, health practitioners were considered more vulnerable than other workers to developing psychological problems and other stress-related disorders, as they treated patients confirmed with COVID-19 while also dealing with misinformation [54].

As noted above, practitioners are recommended to invest in developing high levels of patient trust and to proactively correct health misinformation. However, recommendations presuppose that health practitioners necessarily have the resources to do these things well. Some of the materials produced to educate patients are not always reliable or evidence based, resulting ultimately in a loss of trust on the part of patients [52]. In addition, as noted previously, health practitioners themselves are not necessarily immune from accepting health misinformation as credible. Evidence about the level of knowledge and understanding of COVID-19 among practitioners reveals its unevenness. A study of dentists and oral health practitioners’ knowledge about COVID-19 suggested that their knowledge was at a relatively high level [55]. By contrast, a study of 310 eye care professionals in Nepal revealed some knowledge but also some acceptance of misinformation. Symptoms of COVID-19 were known to 94% of participants, but only 49% of participants were aware of how the disease is transmitted. More significantly, 41% of participants believed that the consumption of hot drinks helps to destroy the virus, in contradiction to World Health Organization information. The mean overall “knowledge” performance score, as measured by the benchmarks set by the researchers, was 69.65% [56].

A qualitative study to investigate primary health care practitioners’ perceptions and understanding of the COVID-19 pandemic was conducted in KwaZulu-Natal, South Africa. The study collected data from 15 participants at 2 different clinics situated in rural KwaZulu-Natal. Participants comprised nurses, physiotherapists, pharmacists, community caregivers, social workers, and clinical associates. Data were collected through individual, in-depth face-to-face interviews using a semistructured interview guide. The participants reported prepandemic and pandemic experiences of fear or denial. There was a perception of poor preparation for the COVID-19 outbreak. The findings also revealed participants’ misperceptions regarding the nature of the COVID-19 pandemic. Researchers concluded that respondents’ misunderstandings regarding the pandemic were primarily a result of misinformation found on social media [57].

The discussion in this section so far has highlighted the significant potential of health practitioners in mitigating the impact of online health misinformation. However, it has also underlined factors that may militate against health practitioners’ ability to do so effectively. Not least of these is the issue of health practitioners’ own knowledge, which coexists with other stressors for health practitioners in combating misinformation. The discussion will now consider health information management (HIM) as a tool for supporting health practitioners’ knowledge base as one element in a multifaceted strategy for combating misinformation on the web.

#### HIM as a Mitigation Strategy

We have seen there is a need for health practitioners to be supported with evidence-based knowledge that they can share with patients. Kyabaggu et al [5] argued that the COVID-19 pandemic has demonstrated that in an infectious health crisis, the gathering of accurate and reliable data to assist with the public health response is essential. They highlighted the importance of HIM professionals in supporting contact tracing and syndromic surveillance, as well as in mapping and forecasting health data. They noted that the generation of health information supports the continuum of care and the setting of targets and indicators and aids the planning, monitoring, and evaluation of health programs locally and globally. The health information produced also underpins the development of equitable, efficient, and accessible health care systems, contributing to improving public health initiatives and outcomes. Kyabaggu et al [5] emphasized the importance of an area of HIM, currently in its early stages, that deals with gathering and identifying evidence about the structural inequalities that underlie the disparities in vulnerability to health misinformation discussed in this paper. The collection of rich, high-quality information, including patient-reported experience, outcome measures, and culturally appropriate identity data, can enable health practitioners and public health advisers serving the most disadvantaged and underrepresented communities to use more tools of advocacy for patients.

The authors noted that advances in technology, including artificial intelligence, have the potential to relieve some of the pressures and constraints on health practitioners working on the front line during crises such as the COVID-19 pandemic, allowing more time for one-to-one engagement with patients. Kyabaggu et al [5] advocated for the content expertise of health information managers to serve health practitioners by delivering patient-facing information triaging services; constructing user-friendly knowledge representations, such as data visualizations; and developing information interpretation tools, such as decision aids, plain language summaries, and supplementary explanatory information and metadata. Kyabaggu et al [5] identified the interdisciplinary underpinnings of HIM as essential in contributing to the educational, informational, and decision-making support for addressing current and future infodemic management crises.

## Discussion

### Summary of Results

Within the literature, there is a consensus that there exists a significant problem of online health misinformation disseminated via the internet on social network platforms, often by online health communities. It is apparent that while users seek trustworthy sources of health information, they are unequally equipped to assess its credibility. This is partly because some groups lack sufficient levels of health and digital literacies, which may be exacerbated by concomitant social and economic inequalities. Reception of, and response to, online health misinformation is also shaped by users’ cultural contexts, values, and experiences, which may hinder trust in scientific institutions and governments. Evidence suggests that some demographics are more vulnerable to accepting health misinformation as credible and that health practitioners are unevenly prepared in the context of new global health crises, such as the COVID-19 pandemic. Furthermore, the evidence of disparities in positive and negative attitudes toward vaccination highlights a need to pay specific attention to regional and national settings, even in the current global context. Preexisting levels of local trust in vaccine providers may be a significant factor to consider. While the validity and reliability of YouGov polls are limited, nevertheless, the data from an admittedly narrow range of sources suggests that vaccine confidence may have become more fluctuating and potentially vulnerable to destabilization in the digital era.

While online mitigation strategies such as user correction and automatic detection may have their uses, their effectiveness is contested, and some studies suggest they may even be counterproductive. Our analysis of the available literature indicates that the effectiveness of these strategies varies and needs further evaluation [42,58]. The issue of online health misinformation is further complicated by the operation of malicious actors and politicization of the issue, particularly during the COVID-19 pandemic, militating against the equitable and trusted dissemination of evidence-based knowledge. The role of health practitioners in this context is a challenging one. Research suggests that on the one hand, they are still best placed, at the front line of care, to combat health misinformation with science-based knowledge and advice. On the other hand, the stressors identified in this review create barriers to their abilities to do this well. Constraints of time and lack of supporting infrastructure add to the knowledge deficit noted earlier. Our review underlines the complexity of the environment in which health practitioners operate and calls for greater support and resources to enable effective mitigation of health misinformation [59]. Investment in HIM at local and global levels could address all 3 deficits, creating the potential for health practitioners to enhance their capacity to build trust via knowledgeable one-to-one communication with patients.

### Limitations

The limitations of this study are the following: First, the constraints of time and space have necessarily limited the scale and scope of the survey. Second, the study of online health misinformation is a growing field, and inevitably, the nature of the issue means that new evidence is emerging at a rapid rate. In particular, new knowledge and further reflection in the wake of the COVID-19 pandemic will continue to shed new light on the subject. Our study acknowledges these limitations and emphasizes the dynamic nature of the field.

### Conclusions

Our survey of the literature on online health misinformation has revealed a complex and multifaceted context in which health practitioners must operate. As the world renormalizes following the pandemic, a collaborative global interdisciplinary effort to provide equitable access to timely, accurate, and complete health information will be needed to support health practitioners in combating the impact of online health misinformation. Academic research will need to be disseminated into the public domain in a way that is accessible to the public to counter misinformation and educate populations concerning how science is carried out. Our conclusions drawn from this review stress the urgency of effective strategies and collaborative efforts to mitigate the prevalence and impact of health misinformation on a global scale. Without strategies for equipping populations with the health and digital literacies required to interpret and use information appropriately, the prevalence of online health misinformation will continue to pose a threat to global public health efforts, disproportionately affecting vulnerable and resource-limited populations. Although social media platforms have a responsibility to correct misinformation, governments will need to engage in evidence-informed decision-making and invest in HIM to support frontline health practitioners in their work, enhance population health literacy, and strengthen evidence-informed decision-making at all levels.

Several issues for further investigation arise from the findings of this review. These include the following:

- The long-term impact of COVID-19 vaccine hesitancy
- Whether the COVID-19 pandemic has intensified or diminished information literacy, and the related question of whether the pandemic will incentivize health information literacy
- The effects of social and cultural differences on the long-term traction of future health misinformation
- Whether social and economic inequalities will become less or more pronounced in the face of a global pandemic
- The comparative effectiveness of strategies to enhance populations’ media and digital literacies to facilitate the mitigation of health misinformation and its effects
- The influence of state actors on the propagation of health misinformation on the web
- The extent to which academic research has been disseminated into the public domain in a way that is accessible to the public, and the effectiveness of strategies to do so to counter misinformation and educate populations concerning how science is carried out

## Appendix

Multimedia Appendix 1 Detailed search strategy.

## Abbreviations

CSM: content similarity measure
ELM: elaboration likelihood model
HIM: health information management
NHS: National Health Service

## Data Availability

The data analyzed in this study are derived from published articles available on Google Scholar. All articles included in the review are cited in the reference list. No additional data or code were collected or generated as part of this study.
