# Supplementary material for: Prevalence of Health Misinformation on Social Media—Challenges and Mitigation Before, During, and Beyond the COVID-19 Pandemic: Scoping Literature Review
Source: J Med Internet Res. 2024 Aug 19;26:e38786. doi: 10.2196/38786 (PMC11369541; doi:10.2196/38786)
Supplement: Multimedia Appendix 1 [file jmir_v26i1e38786_app1.docx]

**Multimedia Appendix 1**

# Detailed search strategy

## Databases and information sources

The databases and sources searched include:

- PubMed
- MEDLINE
- Embase
- Cochrane Library
- Scopus
- Web of Science
- Google Scholar
- Grey literature sources (e.g., conference proceedings, industry reports)

## 2. Search terms and keywords

The search terms and keywords were structured to cover various aspects relevant to the scoping review. The following terms were used:

- Health misinformation
- COVID-19 misinformation
- Vaccine misinformation
- Social media misinformation
- Online health communities
- Information literacy
- Infodemic management
- Misinformation detection
- Misinformation correction
- Public health communication
- Behavioral features
- Machine learning
- Trust in information
- Perception of truth
- Belief in misinformation
- Health professionals
- Job practice impact
- Health literacy
- Equity in health communication
- Psychological impacts
- Message features
- Health disparities
- Spread of misinformation
- Online surveys
- Systematic review
- Troll and bot behavior
- Trusted voices
- Public health agencies
- Cognitive biases
- Debunking misinformation
- Vaccine hesitancy
- Online behavior
- Risk perception
- Narrative framing
- Social network analysis
- Partisan behavior
- Conspiracy theories
- Persuasion techniques
- Information credibility
- Expertise in health communication

## 3. Search strategies

The search strategies were tailored for each database, using Boolean operators (AND, OR, NOT) to combine search terms effectively refine the search results such as: ("health misinformation" OR "misinformation detection") AND ("COVID-19" OR "vaccine misinformation").

| Databases | Terms | Results |
| --- | --- | --- |
| PubMed | ("health misinformation"[Title/Abstract] OR "misinformation detection"[Title/Abstract]) AND ("COVID-19"[Title/Abstract] OR "vaccine misinformation"[Title/Abstract]) AND ("social media misinformation"[Title/Abstract] OR "online health communities"[Title/Abstract] OR "information literacy"[Title/Abstract] OR "infodemic management"[Title/Abstract] OR "misinformation correction"[Title/Abstract] OR "public health communication"[Title/Abstract] OR "behavioral features"[Title/Abstract] OR "machine learning"[Title/Abstract] OR "trust in information"[Title/Abstract] OR "perception of truth"[Title/Abstract] OR "belief in misinformation"[Title/Abstract] OR "health professionals"[Title/Abstract] OR "job practice impact"[Title/Abstract] OR "health literacy"[Title/Abstract] OR "equity in health communication"[Title/Abstract] OR "psychological impacts"[Title/Abstract] OR "message features"[Title/Abstract] OR "health disparities"[Title/Abstract] OR "spread of misinformation"[Title/Abstract] OR "online surveys"[Title/Abstract] OR "systematic review"[Title/Abstract] OR "troll and bot behavior"[Title/Abstract] OR "trusted voices"[Title/Abstract] OR "public health agencies"[Title/Abstract] OR "cognitive biases"[Title/Abstract] OR "debunking misinformation"[Title/Abstract] OR "vaccine hesitancy"[Title/Abstract] OR "online behavior"[Title/Abstract] OR "risk perception"[Title/Abstract] OR "narrative framing"[Title/Abstract] OR "social network analysis"[Title/Abstract] OR "partisan behavior"[Title/Abstract] OR "conspiracy theories"[Title/Abstract] OR "persuasion techniques"[Title/Abstract] OR "information credibility"[Title/Abstract] OR "expertise in health communication"[Title/Abstract])  Filters: Clinical Study, Humans, English, from 2012 - 2024 | 953 |
| Scopus | TITLE-ABS-KEY(("health misinformation" OR "misinformation detection") AND ("COVID-19" OR "vaccine misinformation") AND ("social media misinformation" OR "online health communities" OR "information literacy" OR "infodemic management" OR "misinformation correction" OR "public health communication" OR "behavioral features" OR "machine learning" OR "trust in information" OR "perception of truth" OR "belief in misinformation" OR "health professionals" OR "job practice impact" OR "health literacy" OR "equity in health communication" OR "psychological impacts" OR "message features" OR "health disparities" OR "spread of misinformation" OR "online surveys" OR "systematic review" OR "troll and bot behavior" OR "trusted voices" OR "public health agencies" OR "cognitive biases" OR "debunking misinformation" OR "vaccine hesitancy" OR "online behavior" OR "risk perception" OR "narrative framing" OR "social network analysis" OR "partisan behavior" OR "conspiracy theories" OR "persuasion techniques" OR "information credibility" OR "expertise in health communication"))  AND (LIMIT-TO(DOCTYPE, "ar") OR LIMIT-TO(DOCTYPE, "re") OR LIMIT-TO(DOCTYPE, "cp"))  AND (LIMIT-TO(LANGUAGE, "English"))  AND (LIMIT-TO(SRCTYPE, "j"))  AND (PUBYEAR > 2011 AND PUBYEAR < 2025) | 1047 |
| Web of Science | TS=("health misinformation" OR "misinformation detection")  AND TS=("COVID-19" OR "vaccine misinformation")  AND TS=("social media misinformation" OR "online health communities" OR "information literacy" OR "infodemic management" OR "misinformation correction" OR "public health communication" OR "behavioral features" OR "machine learning" OR "trust in information" OR "perception of truth" OR "belief in misinformation" OR "health professionals" OR "job practice impact" OR "health literacy" OR "equity in health communication" OR "psychological impacts" OR "message features" OR "health disparities" OR "spread of misinformation" OR "online surveys" OR "systematic review" OR "troll and bot behavior" OR "trusted voices" OR "public health agencies" OR "cognitive biases" OR "debunking misinformation" OR "vaccine hesitancy" OR "online behavior" OR "risk perception" OR "narrative framing" OR "social network analysis" OR "partisan behavior" OR "conspiracy theories" OR "persuasion techniques" OR "information credibility" OR "expertise in health communication")  AND PY=(2012-2024)  AND LA=(English) | 1320 |
| Google Scholar | First 10 search-result pages  Go to Google Scholar Advanced Search.  In the "all of these words" field, enter:  "health misinformation" OR "misinformation detection" AND "COVID-19" OR "vaccine misinformation"  In the "at least one of these words" field, enter:  "social media misinformation" "online health communities" "information literacy" "infodemic management" "misinformation correction" "public health communication" "behavioral features" "machine learning" "trust in information" "perception of truth" "belief in misinformation" "health professionals" "job practice impact" "health literacy" "equity in health communication" "psychological impacts" "message features" "health disparities" "spread of misinformation" "online surveys" "systematic review" "troll and bot behavior" "trusted voices" "public health agencies" "cognitive biases" "debunking misinformation" "vaccine hesitancy" "online behavior" "risk perception" "narrative framing" "social network analysis" "partisan behavior" "conspiracy theories" "persuasion techniques" "information credibility" "expertise in health communication"  Set the date range to 2012-2024.  Set the language to English. | 568 |
| MEDLINE | (("health misinformation" OR "misinformation detection") AND ("COVID-19" OR "vaccine misinformation") AND ("social media misinformation" OR "online health communities" OR "information literacy" OR "infodemic management" OR "misinformation correction" OR "public health communication" OR "behavioral features" OR "machine learning" OR "trust in information" OR "perception of truth" OR "belief in misinformation" OR "health professionals" OR "job practice impact" OR "health literacy" OR "equity in health communication" OR "psychological impacts" OR "message features" OR "health disparities" OR "spread of misinformation" OR "online surveys" OR "systematic review" OR "troll and bot behavior" OR "trusted voices" OR "public health agencies" OR "cognitive biases" OR "debunking misinformation" OR "vaccine hesitancy" OR "online behavior" OR "risk perception" OR "narrative framing" OR "social network analysis" OR "partisan behavior" OR "conspiracy theories" OR "persuasion techniques" OR "information credibility" OR "expertise in health communication")).ti,ab. | 246 |
| Embase | #1 'health misinformation'/exp OR 'misinformation detection'/exp  #2 'COVID-19'/exp OR 'vaccine misinformation'/exp  #3 'social media misinformation'/exp OR 'online health communities'/exp OR 'information literacy'/exp OR 'infodemic management'/exp OR 'misinformation correction'/exp OR 'public health communication'/exp OR 'behavioral features'/exp OR 'machine learning'/exp OR 'trust in information'/exp OR 'perception of truth'/exp OR 'belief in misinformation'/exp OR 'health professionals'/exp OR 'job practice impact'/exp OR 'health literacy'/exp OR 'equity in health communication'/exp OR 'psychological impacts'/exp OR 'message features'/exp OR 'health disparities'/exp OR 'spread of misinformation'/exp OR 'online surveys'/exp OR 'systematic review'/exp OR 'troll and bot behavior'/exp OR 'trusted voices'/exp OR 'public health agencies'/exp OR 'cognitive biases'/exp OR 'debunking misinformation'/exp OR 'vaccine hesitancy'/exp OR 'online behavior'/exp OR 'risk perception'/exp OR 'narrative framing'/exp OR 'social network analysis'/exp OR 'partisan behavior'/exp OR 'conspiracy theories'/exp OR 'persuasion techniques'/exp OR 'information credibility'/exp OR 'expertise in health communication'/exp  #4 #1 AND #2 AND #3  Limits: [embase]/lim NOT [medline]/lim AND ('clinical article'/de OR 'human'/de OR 'randomized controlled trial'/de) AND [article]/it AND [humans]/lim AND [english]/lim AND [2012-2024]/lim | 324 |
| Cochrane Library | ("health misinformation" OR "misinformation detection") AND ("COVID-19" OR "vaccine misinformation") AND ("social media misinformation" OR "online health communities" OR "information literacy" OR "infodemic management" OR "misinformation correction" OR "public health communication" OR "behavioral features" OR "machine learning" OR "trust in information" OR "perception of truth" OR "belief in misinformation" OR "health professionals" OR "job practice impact" OR "health literacy" OR "equity in health communication" OR "psychological impacts" OR "message features" OR "health disparities" OR "spread of misinformation" OR "online surveys" OR "systematic review" OR "troll and bot behavior" OR "trusted voices" OR "public health agencies" OR "cognitive biases" OR "debunking misinformation" OR "vaccine hesitancy" OR "online behavior" OR "risk perception" OR "narrative framing" OR "social network analysis" OR "partisan behavior" OR "conspiracy theories" OR "persuasion techniques" OR "information credibility" OR "expertise in health communication") | 84 |
| Grey literature |  | 21 |
| Total |  | 4,563 |
